# Transition to adulthood in Duchenne Muscular Dystrophy: a systematic review with narrative synthesis on health systems, policies, and the role of health care providers

**DOI:** 10.3389/fpubh.2026.1771855

**Published:** 2026-05-29

**Authors:** Sebastian Friedrich, Jana Willems, Sunil Rodger, Jo-Anne Petropoulos, Delaney Ringer, Ellen Wang, Julia Frei, Kinga Pozniak, Anna Swain, Erika Guastafierro, Alessia Marcassoli, Giulia Trucco, Angelica Mazzilli, Gudrun Reeskau, Fernanda De Angelis, Homira Osman, Anne Fournier, Rocio Giselle Gutierrez Rojas, Jan Willem Gorter, Isabella Moroni, Matilde Leonardi, Nardo Nardocci, Olaf Kraus de Camargo, Thorsten Langer

**Affiliations:** 1 Department of Neuropediatrics and Muscle Disorders, Faculty of Medicine, Medical Center, University of Freiburg, Freiburg, Germany; 2 Section of Health Care Research and Rehabilitation Research, Institute of Medical Biometry and Statistics, Faculty of Medicine, Medical Center, University of Freiburg, Freiburg, Germany; 3 McMaster University, Hamilton, ON, Canada; 4 CanChild Center for Childhood-Onset Disability Research, McMaster University, Hamilton, ON, Canada; 5 Neurology, Public Health, and Disability Unit, Fondazione IRCCS Istituto Neurologico Carlo Besta, Milan, Italy; 6 Department of Pediatric Neurosciences, Fondazione IRCCS Istituto Neurologico Carlo Besta, Milan, Italy; 7 German Association for Patients Affected by Muscle Diseases, DGM, Freiburg, Germany; 8 Parent Project aps, Rome, Italy; 9 Muscular Dystrophy Canada, Toronto, ON, Canada; 10 Centre de Recherche du Center Hospitalier Universitaire Sainte-Justine, Montreal, QC, Canada

**Keywords:** Duchenne Muscular Dystrophy, health policy, narrative synthesis, neurodiversity, neuromuscular disease, palliative care, rare diseases, transition to adult care

## Abstract

**Background:**

For youth living with neurodisabilities and rare conditions, transitioning from pediatric to adult care results in significant loss of services and supports. This article examines transition-related health systems, policies and provider roles in the context of Duchenne muscular dystrophy (DMD). DMD is a multi-systemic X-linked disorder mainly characterized by progressive muscle degeneration, with about 30% of patients presenting with neurodevelopmental comorbidities. Due to advances in respiratory and cardiac care, life expectancy has increased significantly, creating a new population of adults living with DMD. This demographic shift has exposed critical gaps in the transition from pediatric to adult health care. To date, there is no systematic review covering existing transition policies and programs. This article utilizes integrated care and continuity of care frameworks to examine transition-related health systems, policies, and provider roles.

**Methods:**

We conducted a PRISMA-compliant systematic review searching OVID Medline, Embase, PsycINFO, CINAHL, Web of Science, and SCOPUS from January 1, 2000, to August 31, 2025. Studies were included if they reported on health systems, programs, policies or health care providers' roles in DMD. For synthesizing evidence, we utilized Popay's Narrative Synthesis framework to analyze health systems, policies, and provider roles across included studies, allowing for an aggregation of a body of heterogenous data (quantitative, qualitative and mixed-methods). This methodological approach ensured that the review moved beyond a simple aggregation of findings to generate new insights into the structural gaps.

**Results:**

42 studies met the inclusion criteria. The programs described in these studies varied from residential life-skills training to respiratory-focused transition protocols. A significant disconnect was identified between international care guidelines and implementation; most initiatives are project-based rather than policy-driven. While neurology is central in pediatric care, respiratory and sleep medicine often become the de facto “medical home” for adults. Crucially, support for patients with neurodiverse development was only discussed in 4 of the 42 studies.

**Conclusion:**

This review underlines a lack of comprehensive care models for DMD transition, specifically within the high-resource settings that dominate the literature. Future policies must bridge the gap between project-based funding and sustainable health systems, specifically addressing neurodiversity and caregiver burden.

## 1 Introduction

### 1.1 Duchenne Muscular Dystrophy as an evolving field

Duchenne muscular dystrophy (DMD) represents one of the most impairing and common pediatric neuromuscular disorders, affecting approximately 1 in 3,500 to 1 in 5,000 live male births. Historically, DMD was considered a pediatric condition; until the late 20th century, survival beyond the second decade of life was rare. The natural history of the disease—characterized by a lack of dystrophin leading to progressive muscle wasting, loss of ambulation by early adolescence, and eventual cardio-respiratory failure—has typically confined the clinical experience of DMD to the realm of pediatric specialists (1).

However, the clinical landscape has undergone a profound transformation. The standardized application of corticosteroid therapy, alongside proactive management of respiratory complications through non-invasive ventilation (NIV) and assisted coughing techniques as well as better cardiologic management and feeding via gastric tubes has altered the disease trajectory. More recent data suggest that a significant proportion of young men with DMD are now living well into their third and fourth decades (2). This epidemiological shift can be considered a sign of biomedical progress, yet it has brought a new challenge in health systems: the emergence of an adult population with complex, multi-systemic needs for whom the adult healthcare sector has only recently begun to prepare (3, 4).

### 1.2 Transition in DMD

The period of adolescence and young adulthood is universally recognized as a time of biopsychosocial flux, characterized by the striving for autonomy, identity formation, and social independence. For young men with DMD, this developmental phase coincides with a notable decline in physical function. Precisely when their peers are gaining independence, patients with DMD often experience increasing dependence on caregivers for activities of daily living, complex technology (e.g., ventilators, power wheelchairs), and care coordination (5).

It is at this critical juncture that the “transition cliff” appears. Pediatric and adult healthcare are organized differently. In pediatric care collaboration between specialists is often times easier to achieve than in adult care. Pediatric care models typically involve a more family-centered approach, often coordinated by a pediatric neurologist or neuromuscular specialist who may have followed the patient since diagnosis. In contrast, adult healthcare models tend to be more disease-centric, emphasizing the patient's own agency to navigate appointments and adhere to treatment plans. For patients with DMD—approximately 30% of whom exhibit neurodevelopmental comorbidities such as autism spectrum disorder (ASD), attention-deficit hyperactivity disorder (ADHD), or obsessive-compulsive disorder (OCD)—the expectation of sudden autonomy in the adult system might add yet another layer of complexity (6).

The challenge of DMD transition is further compounded by the divergence in global health system archetypes. While nationalized systems (e.g., the UK's NHS) may offer centralized guidelines, they often face ‘administrative blockages' between social and medical care (7). In contrast, insurance-based or decentralized systems (e.g., the US or Germany) may provide high-tech interventions but struggle with relational continuity due to fragmented provider networks (8). A comparative perspective is essential to understand how different policy environments facilitate or hinder the implementation of international care standards.

Inadequate transition is not merely an administrative inconvenience; it is a determinant of health outcomes. Poorly managed transition is associated with lapses in medical follow-up, lack of adherence to cardiac and respiratory monitoring, increased emergency department utilization, and a palpable sense of abandonment among patients and families (9). Furthermore, the psychosocial dimensions of transition, e.g., education, employment, independent living, relationships, and sexual health, are frequently overshadowed by the immediate medical imperatives of the disease (10).

### 1.3 Current evidence and theoretical framework: the ideal vs. the reality

To date, few transition programmes in DMD have been described and published in scientific literature, primarily from high-income countries. These single-center studies have described local transition programs. From a health systems perspective, there remains a lack of a comprehensive, systematic synthesis of the global evidence regarding policies for DMD transition. While existing literature has synthesized qualitative patient experiences (5) and clinical milestones post-transition (11), a critical gap remains regarding the structural scaffolding that supports these transitions. Previous reviews primarily adopt an individual-centric lens, focusing on the *phenomenology* of transition (how it feels for the patient) or the *physiology* of transition (clinical outcomes). However, they frequently overlook the policy-level determinants and integrated care models that dictate whether a service is sustainable or merely a time-limited project (12). The current gap in research is a systematic synthesis of these structural components, such as reimbursement frameworks and formal inter-departmental agreements, the ‘transition cliff' remains a descriptive metaphor rather than a target for policy intervention. To lay the ground for policy intervention, a better understanding of structural, not individual, components of transition in DMD is needed. By structural components we mean health systems, their specific policies and the roles of health care providers in transition in DMD. This systematic review aims to fill this gap by focusing specifically on the structures of care.

Increasingly, empirical research underscores that health outcomes in chronic conditions are not merely products of clinical intervention, but are profoundly shaped by environmental and structural determinants (13). In complex health systems, the absence of integrated policy approaches often results in a ‘systemic failure' where clinical progress (e.g., increased life expectancy in DMD) outpaces the structural scaffolding required to support it (14). To address this, current global frameworks emphasize integrated, people-centered health services as a prerequisite for managing the transition from pediatric to adult sectors, moving beyond fragmented, project-based solutions toward sustainable, system-wide strategies (15).

To analyze the current state of transition in DMD, this systematic review draws upon the Continuity of Care framework, which distinguishes between informational continuity (the transfer of medical data), management continuity (consistent clinical protocols), and relational continuity (the ongoing therapeutic relationship) (16). Furthermore, we utilize Integrated Care Models to evaluate how health systems bridge the silos between different specialties and disciplines in pediatric and adult care. Transition is viewed not as a singular administrative event (transfer of files), but as a longitudinal, active process of empowerment and system adaptation (12). It is also examined as a test of a system's ability to maintain integrated policy approaches in the face of multi-systemic complexity. This leads to the tension between two concepts:

The Normative Theory: International DMD care guidelines, specifically the 2018 Lancet Neurology recommendations (17), posit that transition should be a gradual process beginning in early adolescence (ages 12–14). It should involve a multidisciplinary team (MDT), a transition coordinator, and specific milestones for patient education (18).

The Implementation Gap: Despite these guidelines, existing evidence suggests a high degree of heterogeneity in how transition is operationalized globally. Health systems vary in their capacity to provide specialized adult neuromuscular care. In some jurisdictions, patients are “aged out” of pediatric hospitals without a receiving adult specialist; in others, pediatric neurologists continue to see adult patients illicitly to prevent care gaps, a phenomenon known as “failure to launch” (19).

The discrepancy between the normative ideal (comprehensive, gradual, planned transition) and the empirical reality (fragmented, *ad-hoc* transfer) forms the core tension of this review.

### 1.4 Objectives and research question

By employing a narrative synthesis methodology, we aim to summarize the existing landscape of transition in DMD from a health systems point of view. Specifically, the objective of this review is to identify areas for future research and implementation strategies. To do so, we intend to move beyond a simple aggregation of current evidence. Instead, by highlighting gaps between intention and implementation, we seek to provide a roadmap for health care providers and policymakers, to move beyond *ad-hoc* solutions toward sustainable, system-level transition strategies.

The primary Research Questions for this review are:

Which health system frameworks and policies are in place to facilitate transition for patients with DMD in health systems worldwide?

In which ways do health care providers contribute to the different aspects of transition in DMD?

Which future directions should be primarily considered when discussion care structures for transition in DMD?

Specifically, we seek to identify:

1. The types of programs currently reported (e.g., lifestyle-focused vs. medical-focused).
2. The level of implementation (project-based vs. policy-based).
3. The specific medical specialties taking the lead in adult care.
4. The extent to which neurodiversity and caregiver support are integrated into these health system frameworks.

## 2 Methods

### 2.1 Study design and protocol

We conducted a systematic review of the literature to identify and analyze existing frameworks, policies, and health care provider roles regarding the transition from pediatric to adult care for patients with Duchenne Muscular Dystrophy (DMD). Given the anticipated heterogeneity of the data, ranging from government policy documents and clinical guidelines to qualitative assessments of pilot programs and single-center observational studies, a meta-analytic approach was deemed inappropriate. Consequently, we adopted a **Narrative Synthesis** design (20).

This review was conducted in adherence to the **Preferred Reporting Items for Systematic Reviews and Meta-Analyses (PRISMA)** guidelines (21). The protocol focused on identifying structural and systemic elements of transition rather than purely clinical outcomes, aligning with the research question: *Which frameworks and policies are in place to facilitate transition for patients with DMD in health systems worldwide?*

### 2.2 Search strategy and parameters

A comprehensive search strategy was developed to capture the intersection of three key concepts: (1) the specific population (Duchenne and related muscular dystrophies), (2) the phenomenon of interest (transition to adult care), and (3) the structural context (health systems, policies, and programs).

We searched the following six electronic databases:

- OVID Medline
- OVID Embase
- OVID APA PsycINFO
- CINAHL (Cumulative Index to Nursing and Allied Health Literature)
- Web of Science Core Collection
- SCOPUS

#### 2.2.1 Search parameters

The search was limited to studies published between January 1, 2000, and August 31, 2025. The start date was selected to align with the emergence of non-invasive ventilation (NIV) and steroid protocols as standard of care, which began to significantly alter life expectancy and thus necessitate transition planning.

#### 2.2.2 Search terms

Keywords and Medical Subject Headings (MeSH) included combinations of:

- *Population:* “Duchenne Muscular Dystrophy”, “DMD”, “Neuromuscular Disorders”, “Dystrophinopathy”.
- *Transition:* “Transition to Adult Care”, “Transfer of Care”, “Adolescent Health Services”, “Continuity of Patient Care”.
- *Structure/Policy:* “Health Policy”, “Program Evaluation”, “Health Care Delivery”, “Clinical Protocols”, “Models of Care”.

Boolean operators (AND, OR) were utilized to refine results, and truncations were used to capture variations in terminology (e.g., transit^*^ to capture transition, transitioning, transitional). The full search strategy is available as Supplementary Material.

### 2.3 Eligibility criteria and screening

To ensure consistency in screening, we operationally defined our core concepts based on established frameworks in health systems research:

- **Health Policy:** Formal mandates, legislation, or reimbursement frameworks issued by governmental or institutional bodies (e.g., national transition guidelines). This definition encompasses the structural and process-oriented components of health sector reform (22).
- **Programs:** Specific, organized sets of services or interventions implemented at a local or regional level (e.g., the ‘Essen Model'). We viewed programs as complex interventions defined by their functional processes within the health system (23).
- **Provider Roles:** The specific leadership, coordination, or clinical tasks performed by healthcare professionals within the transition process, consistent with international frameworks for interprofessional collaborative practice (24).

Screening was performed in a two-step approach: first, studies were screened based on titles and abstracts, second, remaining studies were screened based on their full-text. Studies were included if they met the following inclusion criteria:

(1) Involved patients with Duchenne and other muscular dystrophies from 15 to 25 years (2) reported on health systems, programs, policies or health care providers' roles.

Studies were excluded if

(1) Article type was a review, a letter or an editorial; (2) they were case studies; (3) involved cells, genetics or animal models; (4) were pharmacological studies; (5) discussed disease characteristics and implications only; (6) addressed muscular and neurological functioning of the disease; (7) the full text was not available (for full-text analysis only); (8) the full text was not available in English, French, Italian, German; (9) patients' and caregivers' experience was discussed, without referring to specific programs, policies or health systems or mentioning health care professionals' roles.

Screening was performed by two independent researchers (SF and DR in for title/abstract screening, SF and EW for full-text screening) and conflicts were resolved by discussion with a third researcher (SR).

### 2.4 Data extraction and synthesis

The methodological quality of included studies was assessed using the JBI Critical Appraisal Tools, selecting the appropriate checklist for each study design (e.g., Checklist for Qualitative Research, Checklist for Analytical Cross-Sectional Studies). Studies were evaluated across key domains including selection bias, data collection integrity, and the congruity between results and interpretation. The full critical appraisal results can be found in Supplementary Table 2.

Data from eligible full-text manuscripts were then extracted by one researcher (SF), to summarize results in the form of a narrative synthesis. Data extraction was supported by two online platforms, covidence.org and elicit.com. Data extraction followed a standardized protocol using a two-step coding process:

1. Structural coding We designed a data extraction table to record data from the studies, which included: general information about the article (journal, year, authors, title), participants information (number and mean age); and study design and assessment tools. Additional study characteristics (Geography, Clinical Focus, Funding Source) were extracted into this summary table.
2. Thematic coding In this form, data were divided into 4 main sections, based on articles' content: type of program, level of implementation, medical specialty or profession involved and role of health care providers. One researcher (SF) performed inductive coding of study findings, identifying recurring themes related to 'Structural Barriers,' 'Provider Shifts,' and 'Sustainability.' These codes were then reviewed by a second researcher (DR) to ensure thematic saturation and interpretative consistency. Conflicts were resolved through consensus-based discussion.

Given the lack of homogenous quantitative outcomes, we applied the Narrative Synthesis framework developed by Popay et al. (20). This approach allows for a rigorous synthesis of diverse evidence types (qualitative, quantitative, and mixed-methods) to tell a coherent “story” about the findings. We operationalized the four elements of the framework as follows:

Element 1: Developing a Theory we utilized the data to construct a theoretical model of how transition is intended to function vs. how it functions in reality. To achieve this, we conceptually distinguished between structural evidence (policy documents, program descriptions) and experiential evidence (qualitative accounts from patients and providers). The latter was not treated as a primary outcome measure but was adopted illustratively to validate the impact of structural gaps on the end-users of the health system. We examined the underlying assumptions in some of the included studies, for example, the assumption that “transfer” of medical records equates to “transition” of care—and contrasted this with the holistic models proposed in guidelines. To avoid circular logic, published guidelines and consensus statements were analyzed as the ‘Normative Framework' (the intended ideal), while empirical studies (chart reviews, surveys) were analyzed as the ‘Observed Reality.' The ‘Implementation Gap' is defined as the analytical distance between these two distinct data sets.

Element 2: Developing a Preliminary Synthesis Data were initially organized through tabulation and clustering. We grouped studies by:

Geography: To identify regional trends (e.g., insurance-based models in the US vs. nationalized health systems in the UK/Europe).

Intervention Type: Grouping studies into ‘Lifestyle/Psychosocial' interventions vs. ‘Medical/Clinical' protocols.

Medical Home: Clustering studies based on which specialty assumed primary responsibility for the adult patient (Neurology vs. Respiratory).

Element 3: Exploring Relationships within and between Studies We analyzed the data to identify factors that explain heterogeneity in transition success using a reciprocal translational analysis approach. We juxtaposed findings from different studies to see if they were consistent or contradictory. When contradictions arose (e.g., success in one region vs. failure in another), we examined contextual moderators such as funding mechanisms or clinic structure. Specifically, we looked for relationships between:

Funding Source and Sustainability: Analyzing whether project-based funding correlated with limited program longevity.

Clinical Phenotype and Transition Pathway: Exploring how the patient's respiratory status (e.g., ventilation dependence) influenced the transition pathway more strongly than their neurological status.

Neurodiversity and Exclusion: Examining patterns how patients with intellectual disabilities were included in standard transition programs.

Element 4: Assessing the Robustness of the Synthesis We critically appraised the included studies to determine the strength of the evidence base. This critical appraisal included noting the scarcity of randomized controlled trials (RCTs) in this domain. Since the objective of this systematic review was not to assess the effectiveness of a specific intervention (e.g., by performing a meta-analysis), but rather to provide an overview of existing evidence, albeit observational, we did not exclude studies from the analysis based on their robustness. Aligning with the objective of investigating the gap between normative ideal and empiric evidence, we assessed the included studies separately between guidelines/recommendations and reports of actual programs. Since many included studies were descriptive reports of programs or observational surveys, we assessed the richness of the data (thickness of description) and the methodological quality using criteria adapted for mixed-methods reviews (plausibility, coherence, and applicability). We weighted our synthesis to reflect the predominance of observational evidence. Consequently, while we can identify recurring structural themes (e.g., the value of a coordinator), the lack of controlled trials prevents us from inferring causality between specific program components and improved long-term survival or quality of life. The findings presented below largely represent ‘expert consensus' and ‘local success stories' rather than empirically validated interventions.

This methodological approach ensured that the review moved beyond a simple aggregation of findings to generate new insights into the structural gaps facing the global DMD community.

## 3 Results

### 3.1 Search results and study characteristics

**Figure 1:**
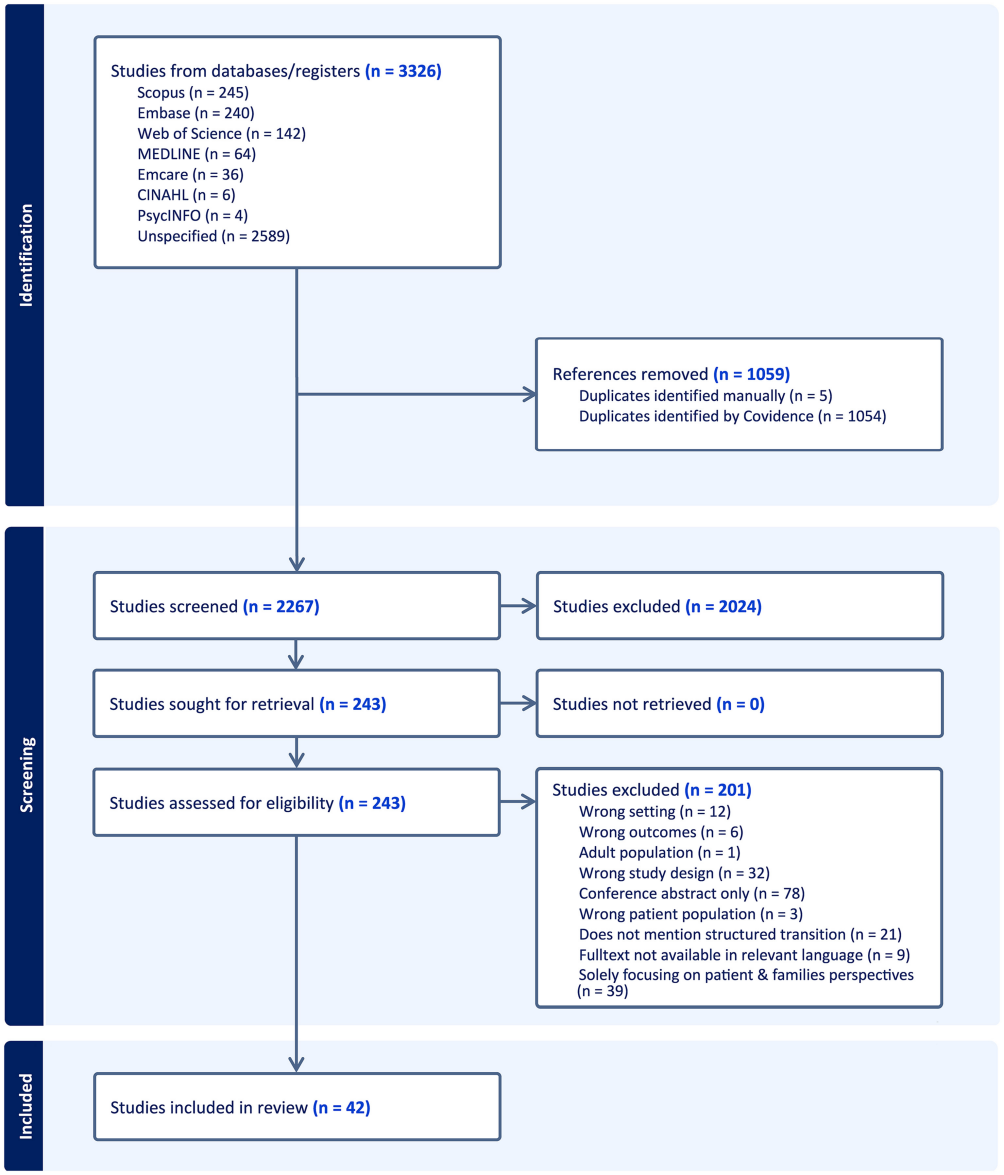
PRISMA flow diagram of the systematic review process.

The systematic literature search yielded a total of 3,326 citations. Following the removal of duplicates and the rigorous PRISMA screening process detailed in Figure 1, 42 studies met the full inclusion criteria. Detailed data extraction was performed on these studies, which provided specific data on health systems, program structures, provider roles, and patient experiences.

The geographic distribution of the included studies reflects a predominance of data from high-income nations with established neuromuscular networks, yet reveals significant regional heterogeneity in care models. The majority of studies originated from Europe (*n* = 9), specifically the United Kingdom (UK), Germany, Italy, the Netherlands, and Romania. North America (*n* = 9) was heavily represented by the United States (US) and Canada. Fewer studies were identified from Oceania (Australia, *n* = 1) and Asia (Hong Kong, *n* = 1; Japan, *n* = 1). Notably, the Delphi consensus study by Molnar et al. (25) provided rare and critical insights into the transition landscape in Central and Eastern Europe, specifically Bulgaria, the Czech Republic, Hungary, Romania, Greece, and Israel, highlighting the resource disparities compared to Western Europe. This geographic skew introduces a significant bias: the transition models described (e.g., Essen, London) are inextricably linked to resource-rich healthcare infrastructures. Their feasibility is often predicated on the availability of high-cost technologies (e.g., CoughAssist™) and reimbursed coordination time, limiting their transferability to the resource-constrained settings identified in the Central and Eastern European studies.

The study designs were predominantly observational, reflecting the nascent stage of transition research in DMD. We identified:

- **Qualitative studies (*n* =** **7):** Utilizing semi-structured interviews and focus groups to explore the lived experience of transition frameworks (e.g., *Hamdani* et al., *Hoskin, Lindsay* et al.).
- **Retrospective chart/case reviews (*n* =** **5):** Analyzing clinical outcomes of specific transition clinics or hospice programs (e.g., *Menon* et al., *Siden* et al., *Onofri* et al.).
- **Surveys and Cross-sectional studies (*n* =** **4):** Assessing provider practices and caregiver perceptions (e.g., *Rodger* et al., *Baldi* et al.).
- **Guidelines and Expert Consensus (*n* =** **4):** Delphi studies defining ‘best practice' (e.g., *Birnkrant* et al., *Castro* et al., *Molnar* et al.).
- **Quality Improvement Projects (*n* =** **1):** Specifically focusing on emergency care planning (*Chouteau* et al.).

A full summary of the characteristics of the included studies is provided in Table 1. A detailed explanation of the characteristics of these studies is provided in Supplementary Table 1.

**Table 1:** Studies included in the systematic review.

No	First author	Year	Title	Journal	DOI
1	Parker A.E.	2005	Analysis of an adult Duchenne muscular dystrophy population	QJM - Monthly Journal of the Association of Physicians	doi: 10.1093/qjmed/hci113
2	Hill, ME	2006	Service provision for adults with long-term disability: a review of services for adults with chronic neuromuscular conditions in the United Kingdom	NEUROMUSCULAR DISORDERS	doi: 10.1016/j.nmd.2005.11.011
3	Camfield P.	2011	Transition to adult care for children with chronic neurological disorders	Annals of Neurology	doi: 10.1002/ana.22393
4	Carter G.T.	2012	Using palliative care in progressive neuromuscular disease to maximize quality of life	Physical Medicine and Rehabilitation Clinics of North America	doi: 10.1016/j.pmr.2012.08.002
5	Rodger, Sunil	2013	The TREAT-NMD care and trial site registry: an online registry to facilitate clinical research for neuromuscular diseases	Orphanet journal of rare diseases	doi: 10.1186/1750-1172-8-171
6	Kinnett K.	2013	Transforming Duchenne Care: Meeting 25-26 June 2012, Ft. Lauderdale, Florida, USA	Neuromuscular Disorders	doi: 10.1016/j.nmd.2013.05.002
7	Schrans, D G M	2013	Transition in Duchenne muscular dystrophy: an expert meeting report and description of transition needs in an emergent patient population: (parent project muscular dystrophy transition expert meeting 17-18 June 2011, Amsterdam, The Netherlands).	Neuromuscular disorders: NMD	doi: 10.1016/j.nmd.2012.08.009
8	Siden H.	2014	Characteristics of a pediatric hospice palliative care program over 15 years	Pediatrics	doi: 10.1542/peds.2014-0381
9	Quinlivan R.	2014	Innovative care model for patients with complex muscle diseases	Current Opinion in Neurology	doi: 10.1097/WCO.0000000000000132
10	Rodger S.	2015	Adult care for Duchenne muscular dystrophy in the UK	Journal of Neurology	doi: 10.1007/s00415-014-7585-3
11	Hamdani, Yani	2015	Transitioning to adulthood with a progressive condition: best practice assumptions and individual experiences of young men with Duchenne muscular dystrophy	Disability and rehabilitation	doi: 10.3109/09638288.2014.956187
12	Lindsay, Sally	2017	Enablers and barriers of men with Duchenne muscular dystrophy transitioning from an adult clinic within a pediatric hospital	Disability and health journal	doi: 10.1016/j.dhjo.2016.10.002
13	Hoskin, Janet	2017	Taking charge and letting go: Exploring the ways a transition to adulthood project for teenagers with Duchenne muscular dystrophy has supported parents to prepare for the future	British Journal of Special Education	doi: 10.1111/1467-8578.12173
14	Schara, U	2018	[Transition from neuropediatrics to neurology in neuromuscular diseases]	Der Nervenarzt	doi: 10.1007/s00115-018-0585-2
15	Trout, Christina J	2018	A Transition Toolkit for Duchenne Muscular Dystrophy	Pediatrics	doi: 10.1542/peds.2018-0333M
16	Birnkrant, David J	2018	Diagnosis and management of Duchenne muscular dystrophy, part 3: primary care, emergency management, psychosocial care, and transitions of care across the lifespan	The Lancet. Neurology	doi: 10.1016/S1474-4422(18)30026-7
17	Anderson J.	2018	Improving service delivery for neuromuscular diseases: a survey of consumers at a tertiary Australian hospital	Internal Medicine Journal	doi: 10.1111/imj.14123
18	Colvin M.K.	2018	Psychosocial management of the patient with Duchenne muscular dystrophy	Pediatrics	doi: 10.1542/peds.2018-0333L
19	Case, Laura E	2018	Rehabilitation management of the patient with Duchenne muscular dystrophy	Pediatrics	doi: 10.1542/peds.2018-0333D
20	Flotats-Bastardas, Marina	2019	[Non-ambulatory patients with Duchenne muscular dystrophy: recommendations for monitoring disease progression and course of treatment]	Der Nervenarzt	doi: 10.1007/s00115-019-0754-y
21	Hiscock, A	2019	'It's a hard conversation to have‘. Healthcare professionals' views concerning advance care discussions with young people affected by life-limiting neuromuscular diseases: an interview study	BMJ SUPPORTIVE & PALLIATIVE CARE	doi: 10.1136/bmjspcare-2017-001369
22	Lindsay, S	2019	Meaningful occupations of young adults with muscular dystrophy and other neuromuscular disorders	CANADIAN JOURNAL OF OCCUPATIONAL THERAPY-REVUE CANADIENNE D ERGOTHERAPIE	doi: 10.1177/0008417419832466
23	Duff C.	2019	Residential immersive life skills programs for youth with disabilities: experiences of parents and shifts in parenting approaches	Journal of Adolescence	doi: 10.1016/j.adolescence.2019.10.015
24	Onofri A.	2019	Transition to adult care in young people with neuromuscular disease on non-invasive ventilation	Italian Journal of Pediatrics	doi: 10.1186/s13052-019-0677-z
25	Wasilewska, Eliza	2020	Transition from childhood to adulthood in patients with Duchenne muscular dystrophy	Medicina (Kaunas, Lithuania)	doi: 10.3390/medicina56090426
26	Chabrol B.	2020	Transition from pediatric to adult care in adolescents with neurological diseases and handicap	Revue Neurologique	doi: 10.1016/j.neurol.2019.09.001
27	Cheng, Pi Chun	2020	Transition of patients with neuromuscular disease and chronic ventilator-dependent respiratory failure from pediatric to adult pulmonary care	Pediatric respiratory reviews	doi: 10.1016/j.prrv.2019.03.005
28	Lu, Mimi	2020	Transition to adult care in sleep medicine.	Pediatric respiratory reviews	doi: 10.1016/j.prrv.2019.09.008
29	Heutinck, Lotte	2021	Clinical Management of Duchenne muscular dystrophy in the Netherlands: barriers to and proposals for the implementation of the International Clinical Practice Guidelines	Journal of neuromuscular diseases	doi: 10.3233/JND-200586
30	Chouteau, Wendy A	2021	Emergency planning as part of healthcare transition preparation for patients with Duchenne muscular dystrophy	Journal of pediatric nursing	doi: 10.1016/j.pedn.2021.08.003
31	Hoskin J.	2021	Troubling norms? Adults and teenagers with a life-limiting impairment in Denmark and England talk about their lives, support and future plans	European Journal of Special Needs Education	doi: 10.1080/08856257.2020.1754545
32	Menon, Deepak	2022	Clinical profile and multidisciplinary needs of patients with neuromuscular disorders transitioning from pediatric to adult care	Neuromuscular Disorders	doi: 10.1016/j.nmd.2021.12.002
33	Taylor, Rachel	2022	Duchenne muscular dystrophy: adult hospice admission survey - doors open or closed?.	BMJ supportive & palliative care	doi: 10.1136/spcare-2022-003997
34	Fleischer, Michael	2022	Essen transition model for neuromuscular diseases	Neurological research and practice	doi: 10.1186/s42466-022-00206-8
35	Cheng H.W.B.	2022	Transition to adult services for young people suffering from life-limiting neurodevelopmental disabilities: a case series	Progress in Palliative Care	doi: 10.1080/09699260.2022.2066270
36	Fleischer, Michael	2023	Essen transition model for neuromuscular diseases.	Der Nervenarzt	doi: 10.1007/s00115-022-01274-6
37	Wollinsky, Kurt	2023	Transition von langzeitbeatmeten Kindern in die Erwachsenenmedizin	Pneumologie	doi: 10.1055/a-2081-0904
38	Molnar, Maria Judit	2024	Essential components of an effective transition from pediatric to adult neurologist care for adolescents with Duchenne muscular dystrophy; a consensus derived using the Delphi methodology in Eastern Europe, Greece and Israel	Orphanet Journal of Rare Diseases	doi: 10.1186/s13023-024-03270-2
39	Spagnoli, Carlotta	2024	Transition and management of patients with Duchenne Muscular Dystrophy: a narrative review based on Italian experts' opinion and real-world experience	Acta Myologica	doi: 10.36185/2532-1900-447
40	Baldi, Olivia	2025	Gaps in the assessment and care of neurodevelopmental and psychiatric conditions associated with dystrophinopathy	Muscle & Nerve	doi: 10.1002/mus.28316
41	Lupu, Maria	2025	Lost in transition: challenges in the journey from pediatric to adult care for a romanian DMD Patient	Healthcare	doi: 10.3390/healthcare13070830
42	Castro, Diana	2025	Transition of patients with Duchenne muscular dystrophy from pediatric to adult care: An international Delphi consensus study	European Journal of Pediatric Neurology	doi: 10.1016/j.ejpn.2025.01.004

### 3.2 Program typologies—The divergence of models

Our narrative synthesis identified a spectrum of transition models ranging from highly formalized medical protocols to *ad-hoc* clinic arrangements. The literature reveals a dichotomy between Medical-Clinical Models, which focus on physical functioning, and Life-Skills Models, which focus on social integration. This dichotomy suggests a fragmentation in service delivery where ‘survival' (medical) and ‘living' (psychosocial) are treated as distinct, potentially competing domains rather than integrated components of a holistic transition strategy. While we acknowledge that some mature programs exhibit hybrid characteristics, studies were categorized based on their primary stated intervention focus (e.g., ventilation management vs. independent living skills) to highlight the distinct theoretical underpinnings of current care approaches.

Interestingly, our results suggest a geographic divergence in transition governance. European models (e.g., Essen, London, Onofri et al. in Italy) lean heavily toward highly structured, hospital-integrated clinical pathways, often leveraging nationalized health infrastructures to maintain medical continuity. Conversely, North American models (e.g., Duff et al. in Canada, Kinnett & Cripe in the US) place a greater emphasis on ‘Life-Skills' and psychosocial enablers, reflecting a decentralized system where transition is often viewed through the lens of individual empowerment and social participation rather than institutional medical handover. An overview of different program typologies is provided in Table 2.

**Table 2:** Comparative table for program typologies.

Model Type	Examples	Primary objective	Governance/ funding	Geographic focus	Performance indicators
Medical-clinical	Essen (GER), London (UK)	Data continuity & survival	Institutional (hospital-led)/state or pharma grants	Europe	Adherence to NIV, Hospital readmissions
Life-skills/immersive	RILS (CAN), Takin' charge (UK)	Autonomy & social integration	Project-based/lottery or charitable grants	North America/UK	Self-efficacy scores, employment rates
Palliative-respite	Canuck place (CAN)	Quality of life & family support	Charitable/health system integration	North America	Days of respite, ACP completion rates

#### 3.2.1 The “Essen Transition Model”: a data-driven medical pathway

Fleischer et al. (26, 27) provided a granular description of the ‘Essen Transition Model' at the University Medicine Essen, Germany. This model represents perhaps the most highly structured, data-driven approach identified in the review (26–28). It is not merely a clinic but a system built upon four distinct pillars designed to prevent the loss of clinical information:

1. Interdisciplinary Standard Operating Procedures (SOPs): The clinic harmonized diagnostic and therapeutic measures across pediatric and adult departments. For example, lung function parameters (vital capacity, cough peak flow) and cardiomyopathy markers were standardized so that an adult neurologist interprets the data exactly as the pediatric neurologist did.
2. Joint Consultations: A defining feature of this model is the overlap period. Young adults do not simply ‘graduate' to adult care; they attend joint consultations involving *both* a pediatric neurologist and an adult neurologist before their 18th birthday. This facilitates a ‘warm handover,' allowing the adult provider to witness the established rapport between the patient and their pediatrician, thereby reducing patient anxiety.
3. The Transition Board: A quarterly interdisciplinary conference brings together pediatric and adult specialists to discuss upcoming transition cases. This ‘board' functions similarly to a tumor board in oncology, ensuring that complex psychosocial or medical issues are flagged before the patient arrives in the adult clinic.
4. The Transition Database: Perhaps the most innovative component is a shared digital infrastructure. The Essen team created a specific database to track longitudinal parameters (BMI, lung function, ejection fraction) across the pediatric-adult divide. This addresses the common ‘data silo' problem where pediatric records are inaccessible to adult providers. It is important to note that this solution was feasible because both pediatric and adult departments operated under the same institutional governance. This contrasts sharply with the ‘administrative blockages' described in UK-based studies (e.g., Abbott & Carpenter), where legal and technical firewalls between pediatric and adult social care services frequently derailed information transfer.

#### 3.2.2 The “London” model: the neuromuscular complex care center (NMCCC)

In contrast to the outpatient-focused Essen model, which attempts to normalize the adult medical home through longitudinal continuity, Quinlivan et al. (2014) described a model centered on elective inpatient admissions at the National Hospital for Neurology and Neurosurgery in London, UK. The NMCCC was designed as a purpose-built, six-bed inpatient unit specifically adapted for adults with severe physical disability (e.g., ceiling hoists, adjusted bathrooms, space for power wheelchairs) (29).

The philosophy of the NMCCC is ‘pre-emptive elective management.' The authors argue that fragmented outpatient care leads to preventable emergencies. Instead of waiting for a crisis (e.g., respiratory failure) to trigger an admission to an acute ward ill-equipped for DMD, patients are admitted for planned ‘MOT' (Ministry of Transport test) assessments. During a short stay (often overnight for sleep studies), patients receive concentrated multidisciplinary input from:

- Respiratory Physicians: For ventilation optimization.
- Cardiologists: For cardiomyopathy management.
- Gastroenterologists: For nutritional support and PEG management.
- Therapists: Physiotherapy, occupational therapy, and speech and language therapy.

This represents a fundamental philosophical divergence: whereas the Essen model relies on the seamless transfer of data within a single university system to prevent care gaps, the London model accepts fragmentation as inevitable, countering it with high-intensity, episodic ‘safety-net' admissions. The NMCCC addresses a barrier identified by Hill et al. (30), and highlighted by Rodger et al. (31) in CARE-NMD survey (30, 31): the physical difficulty and fatigue associated with traveling important distances, e.g., in order to attend multiple outpatient appointments spread across different days. By consolidating care into a ‘one-stop' inpatient episode, the NMCCC reduces the burden on families. However, *Quinlivan* et al. noted that this model requires high staffing ratios (1:2 nursing), making it a resource-intensive solution that may be difficult to replicate in non-specialized centers. Further support for a multi-disciplinary clinic comes from a Canadian study by Menon et al. (32), highlighting the complex healthcare needs of young adults living with neuromuscular conditions (32).

A critical limitation shared by both the Essen and London models is the implicit assumption of patient neurotypicality. Both protocols require a high degree of patient compliance and self-management (e.g., attending appointments, tolerating inpatient stays). Neither study provides specific methodologies for adapting these rigid clinical protocols for the ~30% of DMD patients with significant intellectual disabilities or behavioral comorbidities, a cohort that may struggle with the ‘warm handover' or the sensory environment of a multi-bed inpatient ward.

#### 3.2.3 The immersive life-skills model

Distinct from medical management, Duff et al. (33) evaluated ‘Residential Immersive Life Skills' (RILS) programs in Canada (33). These 1-to-3-week college-based programs are designed to simulate independent living. The study found these programs served as a catalyst for shifting parenting approaches from ‘protective' to ‘autonomy-supportive.' Parents reported that seeing their sons manage daily tasks (directing care, cooking, budgeting) in a safe, supported environment allowed them to visualize a future where their child could live independently.

Similarly, Hoskin (34) evaluated the ‘Takin' Charge' project in the UK. Funded by a lottery grant, this project moved beyond clinical care to address aspirations, employment, and independent living (34). The study highlighted that while the medical system focuses on *survival*, these programs focus on *living*, providing role models and practical skills for budgeting and directing one's own care. The participants in this program reported increased confidence and a shift in mindset from ‘patient' to ‘active citizen.'

Looking at ‘meaningful occupations” for young men living with DMD, Lindsay et al. (34) collected data from patients, families and practitioners via semi-structured interviews. They found that supports and accommodations and self-care skills and coping strategies served as enabler of these kind of occupations. Expectations of a normative adulthood by society, discrimination and inaccessible environments constituted important barriers, as did lack of supports and resources, medical challenges, fatigue, lack of motivation, and social isolation and depression (35).

### 3.3 The structural vacuum—policy vs. projects

A critical finding of this review is the discrepancy between the theoretical ‘ideal' of transition and the reality of funding and policy implementation.

The ‘Implementation Gap': Papers by Birnkrant et al. (1), Castro et al. (18), Chabrol and M. Milh (36), Schrans et al. (37), Trout et al. (38), Wasilewska et al. (39) and Molnar et al. (25) establish a clear global consensus: transition should be a ‘purposeful, planned process' starting in early adolescence (ages 12–14), involving a multidisciplinary team (MDT), and addressing bio-psycho-social needs (17, 18, 25, 36–39). Molnar et al. specifically emphasized, through Delphi consensus, that a ‘Transfer Plan' and ‘Multidisciplinary Transition Summary' are essential documents.

However, the empirical data reveal a ‘structural vacuum.' Rodger et al. (40), analyzing data from the TREAT-NMD Registry, found that over 60% of neuromuscular centers across 42 countries lacked any formal transition arrangements (40). This suggests that for the majority of patients globally, transition remains an *ad-hoc* event rather than a structured process. These findings are echoed by reports from the United States by Kinett and Cripe (2013), illustrating discrepancies in care for children and young adults with DMD (41).

#### 3.3.1 The fragility of project-based care

Most successful programs identified were project-based rather than policy-based, creating sustainability risks:

- The ‘Takin' Charge' program (Hoskin) relied on a lottery grant (34).
- The ‘Essen Model' (Fleischer) was initially funded by a pharmaceutical grant (Sanofi-Genzyme) (26).
- The ‘Canuck Place' hospice (Siden) relied on charitable donations for the majority of its funding (42).

Heutinck et al. (43) explicitly identified ‘lack of funding' as a primary barrier to implementing care guidelines in the Netherlands. They noted that specific roles such as ‘care coordinators' - essential for transition - are rarely reimbursed by standard health insurance models (43). This creates a disincentive for hospitals to formalize transition services, as the time required for coordination is often unbillable.

### 3.4 The medical home—The role of respiratory medicine

The synthesis revealed a potential shift in the ‘primary' medical provider during the transition years. While pediatric care is almost universally led by Neurology/Neuropediatrics (44), the adult ‘medical home' is less defined and often shifts to pulmonology (44, 45). Spagnoli et al. (46) discuss loss of ambulation (LoA) as a potential turning point in DMD care and highlight the increasing importance of cardiac and respiratory care after LoA (46).

Onofri et al. (45) provided a critical comparative analysis of two respiratory units: the Royal Brompton Hospital (London, UK) and Bambino Gesù Children's Hospital (Rome, Italy).

- The UK Cohort: Implemented a structured transition program starting at age 15. By the median age of 17, patients had fully or partially transferred to adult respiratory services.
- The Italian Cohort: Lacked a formal transition pathway. Consequently, 100% of patients (median age 18, ranging up to 22) remained under pediatric respiratory care.
- Clinical Implications: Interestingly, the study found no significant difference in respiratory outcomes (gas exchange, sleep disordered breathing assessment) between the two groups. However, it highlighted that without a dedicated adult respiratory partner, the pediatric system becomes a permanent ‘holding bay' for complex ventilated adults (45).

Cheng et al. (47), Lu et al. (48) and Wollinsky et al. (49) reinforce this, highlighting that for patients with chronic respiratory failure, the pulmonologist is often the only clinician seeing the patient regularly. The management of non-invasive ventilation (NIV), cough assist machines, and secretion management becomes the primary medical necessity for survival (47–49).

Anderson et al. (50) (Australia) offered a contrasting view from the patient perspective. Survey data indicated that only 27% of adult patients supported a new multidisciplinary clinic if it meant losing contact with their ‘usual doctor' (often a single neurologist or respiratory physician). This highlights a tension between the *clinical efficiency* of MDT clinics and the *relational continuity* valued by patients who have spent decades building trust with a single provider (50).

### 3.5 The palliative care landscape—A service mismatch

The review identified a significant disconnect between the potential needs of the adolescent and adult DMD population and the existing adult palliative care infrastructure.

Pediatric Models (Respite and Living): Siden et al. (42) analyzed 15 years of data from *Canuck Place*, a freestanding pediatric hospice in Canada. Neuromuscular diseases constituted the second-largest diagnostic group (20%) after cancer (42). The data revealed that pediatric palliative care (PPC) is utilized ‘for *living*, not dying”.

- 82% of admissions were for scheduled respite care (providing breaks for families).
- Only 7% of admissions were for end-of-life management.
- The median length of stay on the program was 301 days (with many staying years).

Adult Models (The ‘Access Block'): In stark contrast, Taylor et al. (51) surveyed adult hospices in the UK regarding their admission policies (51).

- Admission Disparity: While 98.7% of adult hospices admitted patients with Motor Neurone Disease (MND/ALS), only 42.3% reported admitting patients with DMD.
- The NIV Discrepancy: Crucially, Taylor et al. found that 93.6% of adult hospices admitted patients on NIV. However, qualitative feedback indicated a lack of confidence in managing the *specific* long-term, high-pressure ventilation needs of DMD patients compared to the short-term NIV use in ALS (51).
- Respite Void: Adult hospices generally do not offer the ‘respite' function that families relied upon in the pediatric sector, viewing their mandate strictly as symptom control or end-of-life care.

Hiscock & Barclay (52) noted that healthcare professionals find advance care planning (ACP) discussions significantly harder with young people with neuromuscular conditions than with older cancer patients (52). Cheng et al. (53) demonstrated that a ‘joint care' model (pediatric and adult palliative teams working together for 12–24 months) successfully facilitated ACP, with 89.5% of patients having Do-Not-Attempt-Resuscitation (DNACPR) orders in place, a significantly higher rate than typically seen in *ad-hoc* transitions (53). Chouteau et al. (54) report on a quality improvement project to equip youth with DMD with an individualized emergency care plan (54). Their reports suggest that discussing the matter with patients and families increased their self-perceived knowledge and confidence around potential health emergencies.

### 3.6 The forgotten cohort—Neurodiversity

Colvin et al. (55) and guidelines by Birnkrant et al. emphasize the need for psychosocial management, and Camfield and Camfield (56) provide suggestions on preparing transition for neurodiverse patients and their families (17, 55, 56). Additionally, Case et al. (57) discuss the role of rehabilitation medicine, including the use of assistive technology for neurodiverse patients (57). However, Baldi et al. (6) provide the most granular evidence of the disconnect between patient needs and provider capacity. In their US-based study of 320 caregivers and 74 providers, they found prevalence rates significantly higher than the commonly cited 30%:

- High Prevalence: Caregivers reported high rates of Anxiety (50.5%), ADHD (32.0%), OCD (25.9%), and autism spectrum disorder (21.0%).
- The Assessment Gap: 67.6% of caregivers reported that their primary care provider or neurologist *never* screened for these conditions.
- The Treatment Gap: Less than half of the neuromuscular clinics surveyed had a psychologist or neuropsychologist affiliated with their team. When mental health issues were identified, most neurologists reported referring patients out to general psychiatry services, which are often ill-equipped to handle the intersection of severe physical disability and neurodivergence. This may include, but not be limited to, simple accessibility problems.

Most transition programs described in this review (e.g., the Essen model, RILS) implicitly assume a level of cognitive independence. Lupu et al. (58) used the Transition Readiness Assessment Questionnaire (TRAQ) for a 17-year-old Romanian patient, finding moderate confidence in self-management (58). However, for the ~30% of DMD patients with significant intellectual disability, such tools may be inappropriate, and no specific transition frameworks for this sub-population were identified in the literature.

### 3.7 The lived experience—“Wasting Precious Time”

Qualitative studies provided a necessary counter-narrative to the clinical data, mapping the emotional and social impact of transition.

Lindsay et al. (59) interviewed young men transitioning in Canada (59). Participants described the pediatric setting as ‘warm' and ‘family-centered,' while the adult sector was ‘fragmented,' ‘cold,' and ‘transactional.' One participant poignantly remarked that adult providers seemed to perceive them as ‘dead bodies' rather than young men with futures.

Hoskin (2020) provided a comparative analysis of transition experiences in Denmark vs. England (34).

- **Denmark:** Young men described lives of ‘normality,' supported by state-funded personal assistants (24-h care packages) that allowed them to live independently, pursue education, and socialize freely.
- **England:** Participants described a life of ‘precarity.' Austerity measures and cuts to social care meant that independence was a constant battle. Many lived with parents well into their late 20 s not by choice, but necessity.

Abbott & Carpenter (9) echoed this in their UK-based study titled'*Wasting Precious Time,'* where participants described a post-school existence defined by ‘drift' and a lack of vocational support (9).

Hamdani et al. (60) critiqued the normative goal of ‘independence' itself (60). They noted that for men with DMD, autonomy is inherently ‘interdependent.' Mothers often become de facto care coordinators in adulthood, filling the gaps left by the withdrawal of pediatric services (61).

## 4 Discussion

The results of this systematic review reveal a profound paradox at the heart of Duchenne Muscular Dystrophy (DMD) care. While medical innovation—specifically the widespread adoption of corticosteroid therapy and non-invasive ventilation (NIV)—has successfully transformed DMD from a pediatric life-limiting condition into a somewhat chronic adult disease, care organizations, and health systems globally have failed to keep pace. Our narrative synthesis of 42 studies across five continents indicates that while the'biological” transition to adulthood (i.e., reaching the adult age of 18 years and over) is now a reality for the majority of patients, the *structural* transition of their healthcare remains fragmented, project-dependent, and inequitable.

In this discussion, we apply the third and fourth elements of Popay's narrative synthesis framework—*exploring relationships within the data* and *assessing robustness*—to interpret these findings. We identify the emergence of a ‘Respiratory Paradox' in the medical home, analyze the fragility of project-based care models, and expose the systemic exclusion of neurodiverse patients. We propose the concepts of the ‘Respiratory Paradox' and the ‘Transition Cliff' as original interpretive frameworks to explain why biological survival has not yet translated into structural care continuity.

### 4.1 Popay element 3: exploring relationships and heterogeneity

#### 4.1.1 The “Respiratory Paradox”: the shifting medical home

We term the ‘Respiratory Paradox' as the phenomenon where the ‘medical home' disintegrates upon transfer, necessitating a shift from holistic pediatric neurology to adult respiratory medicine. The following paragraph illustrates how we consider this as a hypothesis-generating construct.

As the neurological progression of the disease reaches a plateau (total loss of ambulation and limited upper limb function), the acute life-threatening risks shift to the cardiorespiratory system. Studies by Parker et al. (62) and Cheng et al. (47) demonstrate that adult engagement with the healthcare system is often driven by the immediate necessity of ventilator maintenance rather than neuromuscular management (47, 62). Lu et al. (48) highlight that for many young adults, the sleep physician becomes the primary point of contact solely because they hold the prescription power for the life-sustaining NIV equipment (48). This shift might be considered problematic for several reasons identified in our review:

1. **Fragmentation of holistic care** While respiratory physicians are experts in ventilation, Anderson et al. (50) note that patients fear losing the ‘whole-person' view provided by their pediatric neurologist. Managing adult care includes managing hypoventilation, cardiac failure, endocrine side effects of long-term steroid use, the orthopedic complications of osteoporosis, or the psychosocial nuances of a progressive impairment (50). This implies the need for coordination, but does not necessarily limit this coordination to adult neurology. Attributing coordination e.g., to pulmonology might necessitate a shift both in training and perceived roles and responsibilities.
2. **The ‘failure to launch'** The data from Onofri et al. (45) illustrates that where a structured adult respiratory pathway does not exist (as seen in their Italian cohort vs. the UK cohort), patients simply do not leave pediatric care. This is not a failure of the patient, but a failure of the system to provide a safe landing. Pediatric neurologists act as a safety net, continuing to treat adults well into their 30s because the alternative, an adult system unprepared for complex neuromuscular disease, is viewed as unsafe (45).

Although the recent Delphi consensus reported by Castro et al. (18) also includes input from adult respirologists, the main conversation in the academic field still seems to happen among neuropediatrics/pediatric neurology and adult neurology (18). We suggest that the potential shift toward respiratory medicine should receive more attention in the years to come.

It is important to distinguish that this ‘paradox' is not an inevitability of the disease, but may rather be a reflection of specific health system incentives. While studies from the UK (25) and US (38) support this shift toward pulmonology-led care, the data from the ‘Essen Model' (15) demonstrates that with specific structural interventions (e.g., joint consultations), the neurological medical home can be preserved. Therefore, the ‘Respiratory Paradox' should be viewed as a system-dependent development rather than a clinical standard.

#### 4.1.2 The governance of precarity: economic & institutional barriers

Our synthesis identified a stark contrast between the robust international care guidelines (1, 18) and the precarious nature of implementation (17, 18). The guidelines call for ‘comprehensive, multidisciplinary transition teams.' Yet, the reality described in the literature is one of project-based precarity.

From a health economics perspective, the current transition landscape is defined by ‘efficiency leaks.' While the pediatric sector invests heavily in intensive corticosteroid and respiratory management, the ‘Transition Cliff' represents a failure to protect that investment. The lack of standardized reimbursement codes for care coordination, highlighted by Heutinck et al. (43) in the Netherlands, creates a disincentive for hospitals to allocate staff to transition roles (43). In terms of governance, most successful programs operate as ‘islands of excellence' supported by short-term grants (e.g., lottery or pharmaceutical sponsorship) rather than as ‘integrated care' models embedded in national policy. This lack of institutionalization leads to high inequality in access, where survival into the third decade is contingent on proximity to a specialized, grant-funded center rather than a universal standard of care.

#### 4.1.3 The neurodiversity gap: absence of specific frameworks

Perhaps the most concerning relationship explored in this synthesis is the correlation between neurocognitive status and transition success. While approximately 30% of patients with DMD present with neurodevelopmental comorbidities, including autism spectrum disorder (ASD), ADHD, and intellectual disability (ID) (6, 55), our review identifies a profound “neuro-normative bias” in existing transition frameworks (6).

Current transition models, such as the Transition Readiness Assessment Questionnaire (TRAQ), operate on a normative assumption of increasing autonomy. The goal is the transfer of decision-making power from the parent to the young adult. However, as Hamdani et al. (60) argue, for a young man with significant intellectual disability, this model of “independent autonomy” is fundamentally flawed and alienating (60). It fails to account for interdependent decision-making, where the caregiver remains a permanent and essential component of the adult care triad.

The “gap” identified by Baldi et al. (6) is not merely clinical; it is institutional and structural (6). Adult neurology services are typically designed for acquired conditions (e.g., stroke, multiple sclerosis) and frequently lack the pediatric expertise or the multidisciplinary time-allocation required to manage developmental neurodiversity. Consequently, these patients face a “double cliff”: they age out of pediatric neurology and pediatric psychiatry simultaneously, often with no receiving adult service capable of managing the intersection of severe physical disability and complex behavioral needs.

From a health systems perspective, the exclusion of this cohort is compounded by the lack of specialized “neuro-transition” pathways. Only 4 of the 42 analyzed studies provided even marginal adaptations for neurodiverse patients. This absence suggests that current transition guidelines, which emphasize self-advocacy and independence, may unintentionally marginalize the most vulnerable 30% of the DMD population. Overcoming this marginalization requires a shift in the research and policy agenda, moving away from “readiness” metrics toward “supported transition” models that integrate psychiatric and behavioral scaffolding into adult neuromuscular clinics. Without such a structural shift, the “warm handover” remains an impossible standard for a significant portion of the DMD community.

However, we must distinguish between active exclusion and the absence of reporting; it is possible that local accommodations exist but are not documented in the literature. Nonetheless, the lack of formalized frameworks remains a critical gap.

Overcoming this marginalization seems even more important looking at examples of successful transition in this area. Weerkamp et al. have recently reported on the home-initiation of nocturnal NIV for two patients with DMD and ADHD or autism spectrum disorder as comorbidity (63).

#### 4.1.4 Correlating results with “integrated care” models

When evaluated against the WHO Framework for Integrated People-Centered Health Services (2016), the transition programs identified in our review reveal a spectrum of integration (15). The “Essen Model” (26) represents a high level of vertical integration, where joint consultations and shared digital infrastructures bridge the hierarchy between pediatric and adult specialties. In contrast, the “London Model” (29) utilizes a hub-and-spoke model of integration, centralizing specialized care to manage the ‘episodic precarity' of adult DMD. However, the ‘Respiratory Paradox' we identified represents a breakdown of functional integration; while informational continuity (data transfer) may occur, management continuity fails as care becomes siloed into pulmonology, losing the holistic oversight required for multi-systemic disease. Our findings suggest that successful transition is not merely a clinical hand-off, but a test of a system's ability to achieve longitudinal integration across a life-course trajectory.

### 4.2 Palliative care: the unspoken transition

A final, crucial theme in our discussion is the integration of palliative care. Transition is not solely about moving to adult curative medicine; it is also about preparing for a life-limiting trajectory. Carter et al. (64) and Siden et al. (42) highlight that palliative care in DMD is not synonymous with ‘end-of-life' care but rather ‘quality-of-life' care (42, 64).

However, Taylor et al. (51) exposed a critical systemic failure: adult hospices are often unprepared for DMD. Unlike pediatric hospices, which are accustomed to respite care for children with complex needs, adult hospices are often geared toward oncology and geriatric care. They may lack the staff capability to manage the CoughAssist^TM^ machines or the complex ventilation regimens of a young man with DMD. This forces young adults to remain in pediatric hospices (where age limits eventually apply) or to lose access to respite and palliative support entirely upon transition. This ‘hospice gap' represents one of the most immediate and distressing failures of the current health system (51).

Also, evidence suggests that discussions around advance care planning and end-of-life decision making are not as frequent as patients would like them to be (10). These discussions could also be continued in adult palliative care settings—but they should start earlier (65, 66).

### 4.3 Structural inequalities in transition

The results of this synthesis underscore profound inequalities in access to services, which can be analyzed across three dimensions of inequity (67):

- **Geographic Inequity:** A stark ‘Global North-South' divide exists. Transition models described in this review are inextricably linked to high-resource infrastructures (e.g., home ventilators, CoughAssist™ machines). In resource-constrained settings, such as those identified in Central and Eastern Europe (25), the primary barrier to transition is not coordination, but premature mortality due to a lack of basic respiratory scaffolding.
- **Cognitive Inequity:** We identified a ‘Neurodiversity Inequity' where transition frameworks are essentially ‘neuro-normative.' Patients with intellectual disabilities are systematically excluded from ‘autonomy-based' transition readiness tools, resulting in a ‘Double Cliff' where they age out of both pediatric neurology and behavioral support simultaneously.

#### 4.3.1 Caregiver-driven inequity

As noted by Hamdani et al. (60), the withdrawal of pediatric support often forces mothers to become ‘de facto care coordinators.' This places a disproportionate economic and gendered burden on families. Access to transition success, therefore, becomes contingent on the family's ‘social capital', their ability to navigate complex adult bureaucracies without institutional support, further widening the gap between high- and low-socioeconomic status households.

### 4.4 Conceptual mapping of the transition landscape in DMD

**Figure 2:**
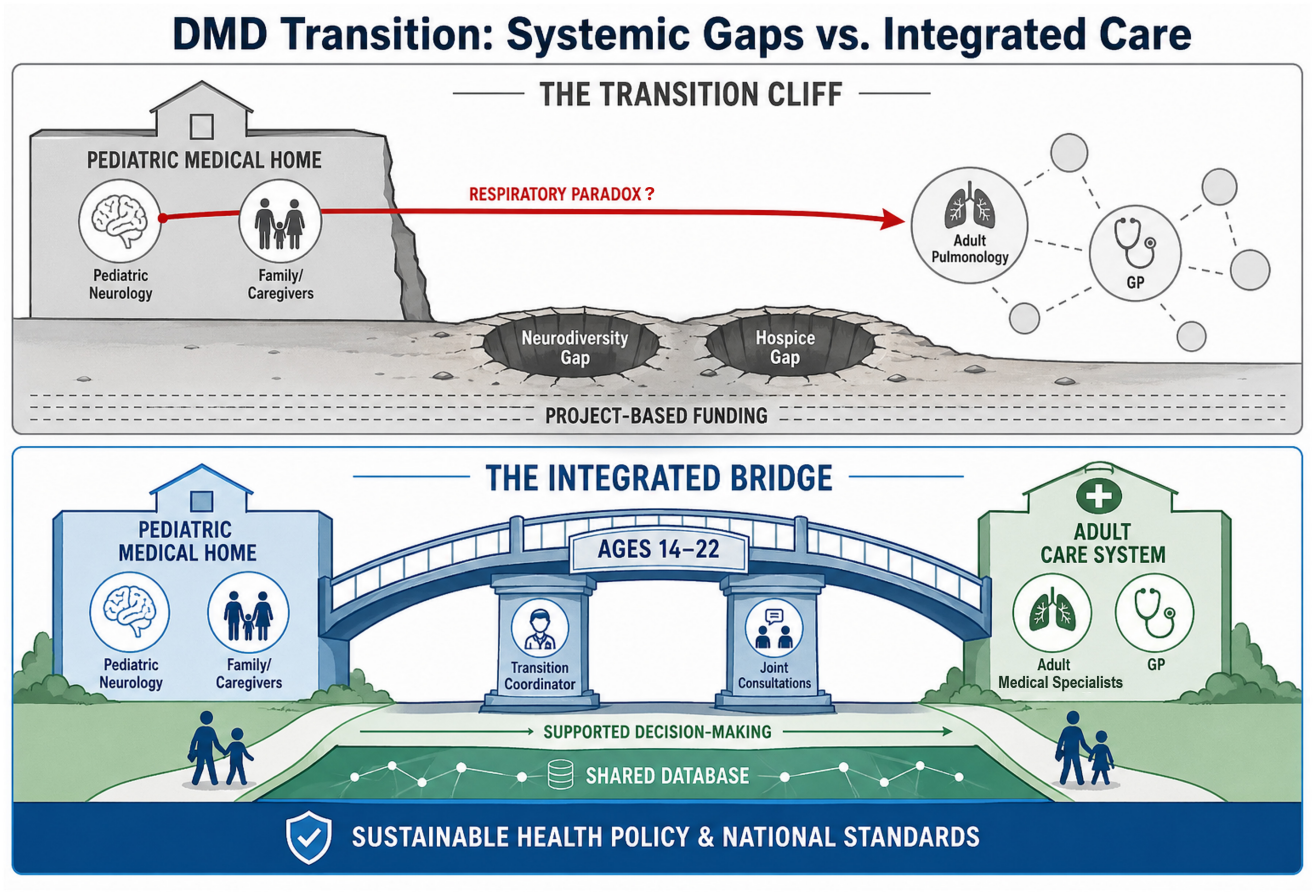
Structural Mapping of the DMD Transition Landscape. The top panel illustrates the “Transition Cliff,” marked by changes such as the hypothesized “Respiratory Paradox”, precarious funding, and systemic gaps in neurodiversity and hospice care. The bottom panel depicts the “Integrated Bridge” model, highlighting a policy-driven framework that utilizes transition coordinators, joint consultations, and shared databases to ensure longitudinal care continuity and supported decision-making.

To synthesize the structural findings of this review and provide a roadmap for policymakers, we propose a conceptual mapping of the DMD transition landscape (Figure 2). This model contrasts the “Observed Reality” of fragmented care with the “Normative Ideal” of a system-level transition.

In the “Transition Cliff” model (Figure 2, Top Panel), the pediatric medical home disintegrates upon transfer, potentially leading to the “Respiratory Paradox” where a more holistic oversight might be replaced by a more specialized approach. The “bumpy road” of transition is characterized by significant systemic “craters”, specifically the “Neurodiversity Gap” and the “Hospice Gap”, where the most vulnerable patients might fall through the cracks of current care structures. Crucially, this model sits on a fragile foundation of “Project-Based Funding,” illustrating how the lack of formal reimbursement for coordination creates a precarious environment that relies on temporary grants rather than sustainable policy.

Conversely, the “Integrated Bridge” (Figure 2, Bottom Panel) illustrates a longitudinal pathway (Ages 14–22) supported by three key structural pillars: a dedicated Transition Coordinator, formal Joint Consultations, and a Shared Digital Infrastructure for data continuity. In this model, the withdrawal of pediatric services is mitigated by a “Supported Decision-Making” framework that keeps families integrated into the adult sector, specifically addressing the needs of neurodiverse adults. This integrated approach is anchored by “Sustainable Health Policy,” moving transition from an *ad-hoc* clinical event to a mandated, institutionally funded governance mechanism.

This conceptual synthesis provides the framework for the following evaluation of the current evidence base and its inherent limitations.

### 4.5 Methodological trends, strengths, and limitations

Our synthesis reveals a critical methodological plateau in the field of DMD transition. The current evidence base is dominated by descriptive, single-center observational studies and expert consensus statements. While these are necessary to identify the problem, the absence of randomized controlled trials (RCTs) or controlled quasi-experimental designs limits our ability to determine causality. We acknowledge that RCTs may not be the gold-standard for assessing transition pathways, as they represent ‘complex interventions”, rather than well-controlled unidimensional ones (68). For now, we know *that* transition programs are associated with better satisfaction, but without control groups and rigorous design of evaluation methodology (quantitative, qualitative or mixed-methods), we cannot isolate which components (e.g., the coordinator vs. the database vs. the joint clinic) drive improved outcomes. Additionally, measuring the quality or outcome of transition in DMD is a challenge in itself, since there is a paucity of patient-reported experience- or outcome-measures in this domain.

The high degree of methodological heterogeneity across the 42 included studies, ranging from international Delphi consensus statements to single-center retrospective chart reviews, precluded a meta-analytic approach. Consequently, the Narrative Synthesis framework was specifically chosen as a robust alternative to mitigate this heterogeneity. By utilizing Popay's four-element model, we were able to move beyond a simple descriptive aggregation of findings. Instead, we generated a conceptual bridge between the ‘Normative Ideal' (guidelines) and the ‘Observed Reality' (local programs), allowing us to identify the structural gaps that a purely quantitative analysis would have overlooked. This methodological choice was essential for a topic where randomized controlled trials are absent, enabling us to construct a holistic picture of the ‘transition landscape' that includes both clinical metrics and structural realities.

Consequently, the strength of the evidence supporting our conclusions varies by theme. The evidence documenting the ‘Implementation Gap' is strong, supported by high concordance across multiple cross-sectional surveys and registry analyses from diverse regions. Similarly, the lack of neurodiversity frameworks is well-documented through consistent gaps in reported protocols. However, conclusions regarding the ‘Respiratory Paradox' rely on retrospective chart reviews from specific tertiary centers, which may reflect local referral patterns rather than a universal natural history. Finally, while the positive impact of Transition Coordinators is a recurring theme, the evidence is primarily qualitative (perceived satisfaction) rather than quantitative (measurable improvement in morbidity or mortality), limiting our ability to make strong causal claims regarding their clinical efficacy.

Another strength of this review is the temporal scope (2000–2025). It captures the entire modern era of Duchenne care, specifically the period following the widespread adoption of long-term ventilation and steroid therapy. This ensures that the review reflects the challenges of the ‘new' adult phenotype rather than historical cohorts with lower survival rates. Last but not least, the inclusion of studies in multiple languages (English, French, Italian, German) and the specific search for recent data from Central and Eastern Europe [e.g., (25)] mitigates some of the Anglo-centric bias often found in medical literature reviews, providing a more nuanced view of how transition functions in varied health systems.

However, several limitations must be acknowledged. First, there is a distinct risk of publication bias favoring successful, well-funded pilot programs. Mature models, such as those in Essen or London, are more likely to be documented in scientific literature because they possess the institutional infrastructure and research funding to support data collection. In contrast, ‘failed' transition initiatives, or clinics where transition is managed through *ad-hoc* crisis admissions without formal protocols, are rarely written up for publication. This suggests that the ‘structural vacuum' identified in our results may, in reality, be even more pervasive than the literature indicates.

Similar to publication bias, there is a risk of survivorship and selection bias: The retrospective chart reviews included [e.g., (32, 62)] inherently suffer from survivorship bias. By definition, these studies analyze patients who successfully navigated the referral pathway to a tertiary adult center. They fail to capture the ‘lost tribe'—the young adults who disengaged from the healthcare system post-pediatrics, those managed solely by general practitioners, or those who died during the care gap. Therefore, our results likely present an overly optimistic view of transition success rates.

Another limitation refers to the geographic and economic bias: Despite efforts to include diverse literature, the evidence base remains heavily skewed toward high-income nations in North America and Western Europe. Data from the Global South (Africa, South America, Southeast Asia) is virtually absent. Consequently, the transition models described—relying on expensive infrastructure like CoughAssist™ machines, home ventilators, and dedicated transition coordinators—may lack applicability in resource-constrained settings where the primary barrier to transition remains premature mortality.

Furthermore, while our synthesis identifies recurring structural themes (e.g., the value of a transition coordinator), the absence of controlled trials prevents us from making statistical claims regarding program efficacy. We can describe *how* a system is structured, but we cannot infer causality between specific structural components and improved long-term survival or quality of life. This review should therefore be viewed as a structural mapping of the transition landscape rather than an empirical validation of clinical interventions. Also, the lack of standardized outcome measures for transition success in DMD makes direct comparison difficult. Some studies defined success as ‘transfer of medical records,' others as ‘attendance at one adult clinic appointment,' and few measured longitudinal adherence or quality of life post-transfer.

As for limitations of the methodology of our review in itself, our search strategy focused on peer-reviewed literature indexed in major databases. Consequently, we may have missed ‘gray literature,' such as unpublished hospital protocols or local government reports, which often contain practical transition policies not described in academic journals. As for language, with inclusion being limited to English, French, German, and Italian likely resulted in the exclusion of relevant data from non-European regions, particularly South America and Asia. This inherently leads to the potential exclusion of reports from low- and middle-income countries.

### 4.6 Future directions: from projects to policy

Based on this synthesis, the direction for future innovation seems to be clear. Moving from ‘pilot projects' and ‘guidelines' toward system-level implementation, guided by implementation science and with robust evaluation designs is a promising approach.

1. Evaluation of coordination Current evidence underlines both a need and a potential for the role of the Transition Coordinator. Currently, one of the major limitations to implementing such a role is the lack of reimbursement. Advocacy for reimbursement requires economic data. Future research could therefore e.g. move toward cost-effectiveness analyses (CEA), comparing the cost of a funded Transition Coordinator against the costs of emergency department visits and ‘failure to launch' admissions associated with *ad-hoc* care (69). This could eventually support establishing a funded position that bridges the pediatric and adult silos.
2. The ‘neuro-respiratory' model To further explore implications of the ‘Respiratory Paradox”, testing shared-care protocols is one possibility: Rather than informal collaboration, researchers should pilot and evaluate formal Service Level Agreements (SLAs) between Neurology and Pulmonology departments. These pilots should measure specific metrics, such as the rate of adherence to follow-up visits, medication or patient-reported outcome measures (70).
3. Neurodiverse-inclusive design Existing transition readiness tools (e.g., TRAQ) are often unsuitable for patients with intellectual disabilities. Future work must focus on developing and validating adapted transition scales (e.g., pictorial or caregiver-assisted versions) to ensure neurodiverse patients are not systematically excluded from transition metrics. This extends to developing ‘interdependent' transition models that formally integrate caregivers into the adult decision-making framework without stripping the patient of their rights to autonomy (71, 72). Also, approaching decision making in healthcare as an interdependent process might be a promising field for future work around transition in DMD (73).
4. Adult neuromuscular training To address the ‘confidence gap' among adult providers, professional societies should develop standardized fellowship rotations or Continuing Medical Education (CME) modules specifically focused on the *adult* phenotype of pediatric neuromuscular diseases, moving beyond the ALS-centric model of adult neuromuscular training.
5. Using the f-words Historically, DMD has been viewed as a ‘neuromuscular condition” and the current organization of care reflects this. Acknowledging DMD as multisystemic disorder is a first step. But integrating more recent understandings of disability and health, such as the International Classification of Functioning (ICF) has the potential to make us move toward discussing transition, life goals and participation—including ‘future”, ‘friends” and ‘fun” (7, 74).

These recommendations are derived from the synthesis of expert consensus and recurring themes in observational data. As they have not been rigorously tested in controlled trials, they should be implemented with accompanying evaluation frameworks.

## 5 Conclusion

The demographic shift in Duchenne Muscular Dystrophy represents a great medical achievement of the 21st century. However, this systematic review reveals that our health systems have not matured alongside their patients. To answer our primary research question—*which health system frameworks and policies are in place to facilitate transition?*—the evidence points to a global landscape defined by fragmentation rather than cohesion. Regarding our objective to identify provider roles, we found a potential shift toward respiratory medicine, and a gap regarding care for a neurodiverse adult DMD population. While we identified innovative models of care, ranging from the structured ‘Essen Transition Model' to the immersive life-skills programs in Canada and the UK, these often remain localized initiatives rather than standardized care pathways.

Distinct from previous reviews that have primarily focused on clinical outcomes or the lived experience of transition, this synthesis uniquely targets the structural determinants of care. We identify some of the drivers behind the discrepancy between pediatric and adult services: the reliance on precarious project-based funding rather than sustainable policy, the misalignment of specialist training with the adult DMD phenotype, and the exclusion of neurodiverse patients from standard care pathways. By aggregating this evidence, we move beyond describing *that* a gap exists to explaining *why* it persists.

Ultimately, our synthesis suggests that transition success is not a clinical variable, but a governance outcome. Without the institutionalization of coordination roles through mandated national standards and sustainable reimbursement frameworks, transition will remain a precarious, project-dependent event rather than a secure component of the lifelong care continuum. While biological survival into adulthood is now common in DMD, the health system remains structurally anchored in a pediatric-only model. To bridge the ‘Implementation Gap,' we propose the following actionable recommendations:

### 5.1 For policy-makers: from guidelines to mandates

- **National Standards:** Policy-makers must move beyond “normative guidelines” toward enforceable national standards. This includes mandating a formal ‘Multidisciplinary Transition Summary' and recognizing the ‘Transition Coordinator' as a designated, reimbursable medical expense. Establishing this structural scaffolding is essential to ensure that transition care does not remain an “unfunded mandate” but becomes a standard, institutionally supported component of rare disease management.
- **Sustainable Funding Scaffolding:** To end the cycle of “project-based precarity,” health systems must shift from temporary, grant-dependent funding toward sustainable ‘Life-Course' funding models. These frameworks must integrate social care, vocational support, and specialized medical respiratory care, providing the long-term institutional stability required to prevent the “Transition Cliff.”

### 5.2 For practitioners and clinic managers: bridging institutional silos

- **Neurodiverse-Inclusive Pathways:** Managers should standardize neurodevelopmental screening within the transition window to identify the ~30% of patients requiring specialized support. Practitioners should move away from neuro-normative “independence” metrics, instead adapting transition tools to support “interdependent agency” and formally integrating caregivers into the adult decision-making framework for non-verbal or cognitively impaired patients.
- **Institutional Governance (SLAs):** To mitigate the “Respiratory Paradox,” clinical leads could establish formal Service Level Agreements (SLAs) between pediatric neurology and adult respiratory departments. These agreements serve as essential governance mechanisms that define roles and responsibilities during the handover period, ensuring that management continuity is maintained and the “medical home” does not disintegrate upon transfer.

### 5.3 Future research agenda: from description to systems analysis

- **Comparative and Interdisciplinary Outcomes:** Future research must pivot from descriptive, single-center pilot studies toward comparative analyses of varied health systems (e.g., contrasting outcomes in centralized vs. decentralized archetypes). Furthermore, there is an urgent need for interdisciplinary research in Health Economics to conduct Cost-Effectiveness Analyses (CEA), providing the financial evidence necessary to advocate for the systemic implementation of transition coordinators and integrated care models.

The era of defining DMD solely as a pediatric condition is over. The challenge now is to build a healthcare system that does not merely keep young men alive, but provides the structural scaffolding to allow them, and their families, to live well. Future research must move beyond describing the problem to rigorously testing the implementation of these systems-level solutions.

## Data Availability

The original contributions presented in the study are included in the article/Supplementary material, further inquiries can be directed to the corresponding author.
