# Supplementary material for: Transition to adulthood in Duchenne Muscular Dystrophy: a systematic review with narrative synthesis on health systems, policies, and the role of health care providers
Source: Front Public Health. 2026 May 29;14:1771855. doi: 10.3389/fpubh.2026.1771855 (PMC13260157; doi:10.3389/fpubh.2026.1771855)
Supplement: Supplementary file 1 [file Data_Sheet_1.docx]

Transition in Duchenne Muscular Dystrophy: A Systematic Review with Narrative Synthesis on Health Systems, Policies, and the Role of Health Care Providers

**Sebastian Friedrich^1^*, Jana Willems^2^, Sunil Rodger^1^, Jo-Anne Petropoulos^3^, Delaney Ringer^3^, Ellen Wang^3^, Julia Frei^3^, Kinga Pozniak^4^, Anna Swain^4^, Erika Guastafierro^5^, Alessia Marcassoli^5^, Giulia Trucco^6^, Angelica Mazzilli^5^, Gudrun Reeskau^7^, Fernanda De Angelis^8^, Homira Osman^9^, Anne Fournier^10^, Rocio Giselle Gutierrez Rojas^10^, Jan Willem Gorter^4^, Isabella Moroni^6^, Matilde Leonardi^5^, Nardo Nardocci^6^, Olaf Kraus de Camargo³, Thorsten Langer^1,4^**

^1^Clinic of Neuropediatrics and Muscle Disorders, Faculty of Medicine, Medical Center, University of Freiburg, Freiburg, Germany.
^2^Section of Health Care Research and Rehabilitation Research, Institute of Medical Biometry and Statistics, Faculty of Medicine, Medical Center, University of Freiburg, Freiburg, Germany.
^3^ McMaster University, Hamilton, Canada ^4^ CanChild Centre for Childhood-Onset Disability Research, McMaster University, Hamilton, Canada
^5^Neurology, Public Health, and Disability Unit, Fondazione IRCCS Istituto Neurologico Carlo Besta, Milan, Italy
^6^Department of Pediatric Neurosciences, Fondazione IRCCS Istituto Neurologico Carlo Besta, Milan, Italy
^7^German Association for Patients affected by Muscle Diseases, DGM, Freiburg, Germany
^8^Parent Project APS, Rome, Italy
^9^Muscular Dystrophy Canada, Toronto, Canada
^10^Centre de Recherche du Centre Hospitalier Universitaire Sainte-Justine, Montreal, Canada

**Correspondence:**Sebastian Friedrich
sebastian.friedrich@uniklinik-freiburg.de

Keywords: Duchenne muscular dystrophy; Transition to adult care; Health policy; Narrative synthesis; Neurodiversity; Palliative care; Neuromuscular disease, Rare Diseases.

**Full Systematic Review’s Search String**

Database: OVID Medline Epub Ahead of Print, In-Process & Other Non-Indexed Citations, Ovid MEDLINE(R) Daily and Ovid MEDLINE(R) 1946 to Present

Search Strategy:

--------------------------------------------------------------------------------

1 Muscular Dystrophy, Duchenne/

2 (Pseudohypertrophic Childhood Muscular Dystrophy or Duchenne or Pseudohypertrophic Muscular Dystrophy or Becker* Muscular Dystrophy or DMD or "pseudo hypertrophic myopathic progressive muscular dystrophy").mp.

3 1 or 2

4 Transition to Adult Care/

5 ("handover to adult" or "hand-over to adult" or "hand-off to adult" or "handoff to adult" or "transfer* from p?ediatric to adult care" or "transfer from adolescen* to adult care" or "transfer* to adult care" or "transfer* to adult oriented care" or " transferred out of the child health system" or "primary-care transfers for youth?" or "primary-care transfers for adolescen*" or "primary-care transfers for children" or "primary-care transfers for p?ediatric?" or "primary care transfers for teen*").mp.

6 ((transfer? or transferred or transferral or transferring) and (adult? adj3 (care or service? or center? or centre? or clinic? or facility or facilities or unit? or department* or hospital? or intrahospital? or intra-hospital? or interfacilit* or inter-facilit*))).mp.

7 ((transfer? or transferred or transferral or transferring) adj10 (care or service? or center? or centre? or clinic? or facility or facilities or unit? or department* or patient? or p?ediatric? or hospital? or intrahospital? or intra-hospital? or interfacilit* or inter-facilit*)).mp.

8 Transitional care/

9 transition*.mp.

10 (shared adj3 (care or service*)).mp.

11 "continuity of patient care"/ or patient handoff/

12 (patient adj3 (hand-off* or handoff*)).mp.

13 (continu* adj3 care).mp.

14 Patient Care Planning/

15 (care adj2 planning).mp.

16 "Delivery of Health Care, Integrated"/

17 ("integrated delivery system?" or "integrated health care system?" or "integrated healthcare system?").mp.

18 or/4-17

19 3 and 18

20 limit 19 to yr="2000 - Current"

***************************
