# Supplementary material for: Transition to adulthood in Duchenne Muscular Dystrophy: a systematic review with narrative synthesis on health systems, policies, and the role of health care providers
Source: Front Public Health. 2026 May 29;14:1771855. doi: 10.3389/fpubh.2026.1771855 (PMC13260157; doi:10.3389/fpubh.2026.1771855)
Supplement: Supplementary file 3 [file Table_2.docx]

Supplementary Table 2:

JBI (Joanna Briggs Institute) Critical Appraisal for all 42 manuscripts included in the review.The following specific JBI checklists were applied:

- **Textual/Opinion (TO):** For guidelines, expert consensus, and descriptive meeting reports (6 questions).
- **Qualitative (Q):** For interviews, focus groups, and phenomenological studies (10 questions).
- **Analytical Cross-Sectional (ACS):** For surveys and registry data (8 questions).
- **Case Series (CS):** For retrospective chart reviews and clinical profiles (10 questions).
- **Case Report (CR):** For single-patient longitudinal descriptions (9 questions).

**Table 2: JBI Quality Assessment of Included Studies**

| **No** | **First Author** | **Year** | **Study Design Type** | **JBI Checklist** | **Score (Met/Total)** | **Quality Rating** |
| --- | --- | --- | --- | --- | --- | --- |
| 1 | Parker A.E. | 2005 | Population Analysis | CS | 8/10 | High |
| 2 | Hill, M.E. | 2006 | Services Review | TO | 5/6 | High |
| 3 | Camfield P. | 2011 | Narrative Review | TO | 6/6 | High |
| 4 | Carter G.T. | 2012 | Clinical Review | TO | 4/6 | Medium |
| 5 | Rodger, Sunil | 2013 | Registry Analysis | ACS | 7/8 | High |
| 6 | Kinnett K. | 2013 | Meeting Report | TO | 4/6 | Medium |
| 7 | Schrans D.G.M | 2013 | Expert Consensus | TO | 6/6 | High |
| 8 | Siden H. | 2014 | Hospice Case Review | CS | 9/10 | High |
| 9 | Quinlivan R. | 2014 | Innovative Model | TO | 5/6 | High |
| 10 | Rodger S. | 2015 | Survey (UK) | ACS | 7/8 | High |
| 11 | Hamdani, Y. | 2015 | Qualitative (Experience) | Q | 9/10 | High |
| 12 | Lindsay, S. | 2017 | Qualitative (Barriers) | Q | 10/10 | High |
| 13 | Hoskin, J. | 2017 | Program Evaluation | Q | 8/10 | Medium |
| 14 | Schara, U. | 2018 | Clinical Protocol | TO | 5/6 | High |
| 15 | Trout, C.J. | 2018 | Toolkit/Guidelines | TO | 6/6 | High |
| 16 | Birnkrant, D. | 2018 | Int. Care Guidelines | TO | 6/6 | High |
| 17 | Anderson J. | 2018 | Consumer Survey | ACS | 6/8 | Medium |
| 18 | Colvin M.K. | 2018 | Psychosocial Review | TO | 5/6 | High |
| 19 | Case, L.E. | 2018 | Rehab Management | TO | 5/6 | High |
| 20 | Flotats-Bastardas | 2019 | Clinical Recommendations | TO | 6/6 | High |
| 21 | Hiscock, A. | 2019 | Qualitative (ACP) | Q | 9/10 | High |
| 22 | Lindsay, S. | 2019 | Qualitative (Occupation) | Q | 9/10 | High |
| 23 | Duff, C. | 2019 | Qualitative (RILS) | Q | 8/10 | Medium |
| 24 | Onofri, A. | 2019 | Case Series (NIV) | CS | 8/10 | High |
| 25 | Wasilewska, E. | 2020 | Case Series (Lithuania) | CS | 7/10 | Medium |
| 26 | Chabrol, B. | 2020 | Transition Review | TO | 5/6 | High |
| 27 | Cheng, P.C. | 2020 | Pulmonary Review | TO | 5/6 | High |
| 28 | Lu, M. | 2020 | Sleep Med Review | TO | 5/6 | High |
| 29 | Heutinck, L. | 2021 | Qualitative (Barriers) | Q | 9/10 | High |
| 30 | Chouteau, W.A. | 2021 | Qualitative (Emergency) | Q | 7/10 | Medium |
| 31 | Hoskin, J. | 2021 | Qualitative (Comparative) | Q | 9/10 | High |
| 32 | Menon, D. | 2022 | Case Series (Adult Care) | CS | 8/10 | High |
| 33 | Taylor, R. | 2022 | Survey (Hospice) | ACS | 7/8 | High |
| 34 | Fleischer, M. | 2022 | Case Series (Essen) | CS | 9/10 | High |
| 35 | Cheng, H.W.B. | 2022 | Case Series (Palliative) | CS | 8/10 | High |
| 36 | Fleischer, M. | 2023 | Program Update | TO | 5/6 | High |
| 37 | Wollinsky, K. | 2023 | Ventilation Protocol | TO | 5/6 | High |
| 38 | Molnar, M.J. | 2024 | Delphi Study (EE) | TO | 6/6 | High |
| 39 | Spagnoli, C. | 2024 | Narrative Review | TO | 5/6 | High |
| 40 | Baldi, O. | 2025 | Survey (Neurodiversity) | ACS | 8/8 | High |
| 41 | Lupu, M. | 2025 | Case Report (Romania) | CR | 9/9 | High |
| 42 | Castro, D. | 2025 | Delphi Study (Int.) | TO | 6/6 | High |
